# Investigating the effect of Icaritin on hepatocellular carcinoma based on network pharmacology

**DOI:** 10.3389/fphar.2023.1208495

**Published:** 2023-05-30

**Authors:** Zhong Xue, Fan Zhang, Shaohua Xu, Minyong Chen, Mingzuo Wang, Ming Wang, Fayong Ke, Zhaoshuo Chen, Mingji Zhang

**Affiliations:** Department of Hepatobiliary and Pancreatic Surgery, Clinical Oncology School of Fujian Medical University, Fujian Cancer Hospital, Fuzhou, China

**Keywords:** hepatocellular carcinoma, Icaritin, multi-omics, pharmaco-omics, proteomics, FYN gene, molecular mechanisms

## Abstract

Hepatocellular carcinoma is one of the cancers that kill people in the global population. Icaritin, a small molecule drug approved by NMPA, has demonstrated potential anti-HCC effects. However, its underlying molecular mechanisms remain unclear. We employed a multi-omics approach in this study, including pharmaco-omics and proteomics, to look into the Icaritin’s possible molecular targets and workings in the therapy of HCC. Through pharmaco-omics analysis, we identified ten putative target genes of Icaritin, including FYN. The relationship between Icaritin and these target genes, particularly FYN, was further validated through *in vitro* and *in vivo* experiments. The outcomes revealed that Icaritin may exert its anti-HCC effects through modulating the FYN gene, highlighting the importance of multi-omics approaches in drug discovery research. This research gives valuable insights regarding the therapeutic potential of Icaritin against HCC and its possible molecular mechanisms.

## 1 Introduction

Hepatocellular carcinoma (HCC) has been increasing in incidence and is the most frequent primary liver cancer and the third greatest cause of cancer-related deaths globally (Villanueva, 2019; Benson et al., 2021). Despite significant advances in early diagnosis and various options for treatment, the prognosis of patients with HCC remains bleak because of its high recurrence and metastasis rates (Llovet et al., 2021). Therefore, there is a pressing need for novel therapeutic strategies to combat HCC.

Icaritin, a small molecule compound approved by the NMPA, has gained increasing attention for its extensive anti-tumor effects (ZENG, 2020; Zeng, 2022). Accumulating evidence has revealed that Icaritin possesses diverse pharmacological properties, such as anti-tumor, estrogen-like activity, osteogenesis, and neuroprotection (Ye and Lou, 2005; Huang et al., 2007; Wang et al., 2007; Zhang et al., 2020). Numerous preclinical studies and clinical trials have been conducted to investigate the potential therapeutic applications of Icaritin in various diseases, including cancer. In particular, Icaritin has shown promise in the treatment of hepatocellular carcinoma (HCC), with *in vitro* and *in vivo* studies demonstrating a strong inhibitory effect on the growth of HCC cells. However, the precise molecular mechanism underlying its anti-HCC activity warrants further investigation (Liu et al., 2022).

To investigate the potential molecular mechanisms of Icaritin’s action against HCC, we propose employing a multi-omics approach, combining pharmaco-omics and proteomics, to explore the interaction between the drug and the biological system (Nogales et al., 2022). Pharmaco-omics encompasses various disciplines, including pharmacogenomics, pharmacoproteomics, and pharmacometabolomics, which together allow for a holistic understanding of drug action and response. This integrative approach will enable us to elucidate the therapeutic targets and pathways involved in Icaritin’s anti-tumor activity.

In addition to pharmaco-omics, proteomics plays a critical role in understanding the molecular mechanisms underlying drug action. Proteomics involves the large-scale analysis of proteins, their structures, functions, and interactions within a biological system. By examining the proteomic profile of HepG2 cells treated with Icaritin, we can identify key protein alterations that maybe helpful for the anti-HCC action of this compound. Combining pharmaco-omics and proteomics will provide a comprehensive view of the molecular mechanisms by which Icaritin exerts its anti-HCC activity.

For present research, our aim is to meticulously research the targeting and molecular mechanisms of Icaritin on HCC using a multi-omics approach, integrating pharmaco-omics and proteomics. This comprehensive investigation will enable us to better understand the therapeutic targets and pathways involved in Icaritin’s anti-tumor activity and provide insights for future research and drug development.

## 2 Materials and methods

### 2.1 Construction of pharmaco-omics network

#### 2.1.1 Data collection and processing

The chemical structure of Icaritin was obtained from PubChem (https://pubchem.ncbi.nlm.nih.gov/). Potential targets of Icaritin were collected from SwissTargetPrediction (http://www.swisstargetprediction.ch/), PharmMapper (http://www.lilab-ecust.cn/pharmmapper/), and TargetNet databases (http://targetnet.scbdd.com/), focusing on *Homo sapiens* targets (Yao et al., 2016; Wang et al., 2017; Daina et al., 2019). After removing duplicates, the potential targets were imported into the UniProt database (https://www.uniprot.org/) and converted to UniProt IDs with corresponding gene names.

#### 2.1.2 HCC disease targets collection

Hepatocellular carcinoma (HCC) related targets were gathered using the GeneCards database (https://www.genecards.org/), with “hepatocellular carcinoma” and “liver cancer” as keywords (Safran, 2022). Duplicates were removed, and target names were normalized using the UniProt database (https://www.uniprot.org/).

#### 2.1.3 Protein-protein interaction (PPI) analysis

Intersection of Icaritin targets and liver cancer disease targets were performed using R software, yielding the anti-HCC targets of Icaritin. The anti-HCC targets of Icaritin were uploaded to the STRING database (https://string-db.org/) to obtain PPI information. A confidence score of ≥0.9 was set for interactions, and unconnected targets were discarded, resulting in a PPI network.

#### 2.1.4 Identification of core targets

The core targets (hub genes) were screened using the cytoHubba plugin in Cytoscape (https://cytoscape.org/), utilizing the Maximal Clique Centrality (MCC) method. The top ten core targets were visualized, with darker colors indicating higher scores and importance within the network.

#### 2.1.5 Enrichment analysis

The DAVID tool was employed to do an enrichment study on the anti-HCC targets of Icaritin (Sherman et al., 2022). This included Gene Ontology (GO) analysis and Kyoto Encyclopedia of Genes and Genomes (KEGG) analysis, with GO analysis covering cellular components (CC), molecular functions (MF), and biological processes (BP).

#### 2.1.6 Molecular docking

The X-ray crystal structures of core targets were obtained from the Protein Data Bank (PDB) (https://www.rcsb.org/), while Icaritin’s structure file was obtained from PubChem (https://pubchem.ncbi.nlm.nih.gov/) and optimized using Chem3D. Protein receptor and ligand files were processed with AutoDock tools (http://autodock.scripps.edu/), followed by molecular docking of Icaritin to core targets using AutoDock Vina software (https://vina.scripps.edu/) (Morris et al., 2009; Trott et al., 2009). The lowest binding energy binding mode was selected for further analysis, and interactions were visualized as 2D plots using PyMOL software (https://pymol.org/2/).

#### 2.1.7 Molecular dynamics simulation

Molecular dynamics simulations were carried out for 20 ns on selected protein-ligand complexes to determine binding affinity (Que et al., 2021). And utilizes the GROMACS 2021 software running on Linux Ubuntu 20.04, driven by an AMD R5 3600 CPUU (Hess et al., 2008). Generation of protein topology using Charmm36 force field and construction of ligand topology using CGenFF (Vanommeslaeghe et al., 2012; Vanommeslaeghe and MacKerell, 2012). The TIP3P water model was employed for solvation, and the system was neutralized with Na+ and Cl- (Hess, 2008). Energy minimization, heating, and equilibration steps were performed, followed by a 20 ns molecular dynamics production run. Trajectories were used for kinetic studies and protein-ligand free energy calculations with MM/PBSA (Paissoni et al., 2014).).

### 2.2 Proteomic

#### 2.2.1 Cell culture and treatment

HepG2 human hepatocellular carcinoma cells were cultured in Dulbecco’s Modified Eagle’s Medium (DMEM) obtained from Gibco (catalog #11965-092), supplemented with 10% fetal bovine serum (FBS) from Hyclone (catalog #SH30396.03), 100 U/mL penicillin, and 100 μg/mL streptomycin from Invitrogen (catalog #15140-122). Cells were maintained in a humidified atmosphere containing 5% CO_2_ at 37°C. For treatment, cells were seeded in 6-well plates at a density of 1 × 10^5 cells per well and allowed to adhere overnight. The cells were then treated with either vehicle (DMSO) or Icaritin (10 μM, Sigma, catalog SML0551) for 2 days. Each treatment group consisted of three replicates, including the control group.

#### 2.2.2 Colony formation assay

For the colony formation assay, cells were seeded into 6-well plates at a density of 500 cells per well and allowed to adhere for 24 h. Following this, cells were treated with 10 µM Icaritin for 2 days. After the treatment, the medium was replaced with fresh drug-free medium, and the cells were allowed to grow for an additional 14 days to form colonies. The medium was changed every 3 days to maintain optimal growth conditions. Once the colonies were clearly visible, the cells were washed twice with cold PBS, fixed with 4% paraformaldehyde from Sigma-Aldrich (catalog #158127) for 15 min at room temperature, and stained with 0.1% crystal violet solution from Sigma-Aldrich (catalog #C3886) for 30 min. Excess stain was washed away with distilled water, and the plates were allowed to air-dry. Colonies consisting of 50 or more cells were counted, and the colony formation efficiency was calculated as the number of colonies divided by the number of cells seeded, expressed as a percentage. The experiment was performed in triplicate, and the results were averaged to obtain the final colony formation efficiency.

#### 2.2.3 Studies involving animal subjects

The Animal Ethics Committee of Fujian Medical University (Fuzhou, China) approved the animal experiment under IACUC FJMU 2022-0036. Six-week-old male BALB/c nude mice were utilized for this investigation. Prior to the study, the animals were housed in a specific pathogen-free facility, under regulated temperature and lighting conditions, with unrestricted access to food and water. HepG2 cells were prepared by harvesting and suspending them in sterile phosphate-buffered saline (PBS) from Gibco (catalog #10010-023) at a suitable concentration for injection. Mice were then inoculated subcutaneously with HepG2 cells in the right flank region. Following tumor establishment, the mice were randomly allocated into two groups: the Icaritin treatment group (*n* = 6) and the control group (*n* = 6). Mice in the Icaritin treatment group received intraperitoneal injections of Icaritin at a concentration of 0.2 mg/kg Icaritin, while those in the control group were given injections of physiological saline. Injections were administered for 8 weeks until the study’s completion. Tumor progression and body weight were periodically assessed. At the end of the experiment, mice were euthanized, and tumor tissues were excised for subsequent analysis.

#### 2.2.4 Proteomics analysis of tumor tissues

For a proteomics study, tumor samples from the Icaritin therapy group and the control group were gathered. After being homogenized, the tissues were lysed in lysis solution that included protease inhibitors. The BCA protein assay was used to determine the protein concentrations. Equal amounts of protein were separated by sodium dodecyl sulfate-polyacrylamide gel electrophoresis (SDS-PAGE) and subjected to in-gel digestion. Each sample had the same quantity of proteins removed for reduction, alkylation, and trypsin digestion. Identification by tandem mass spectrometry (LC-MS/MS) analysis and quantify peptides using an Orbitrap mass spectrometer coupled to a nano-flow liquid chromatography system. On a reverse-phase C18 column, peptides were isolated and then analyzed using data-dependent acquisition mode. MaxQuant software was applied to analyze the raw mass spectrometry data for label-free protein identification and quantification. A false discovery rate (FDR) of 1% was applied to filter the identified proteins.

#### 2.2.5 Quantitative real-time polymerase chain reaction (qPCR) analysis

Total RNA from tumor tissues and cell lines was extracted using TRIzol reagent (Invitrogen, #15596026) according to the manufacturer’s instructions. The concentration and purity of the isolated RNA were determined using a NanoDrop spectrophotometer (Thermo Fisher Scientific). Complementary DNA (cDNA) was synthesized from the total RNA using a reverse transcription kit (Thermo Fisher Scientific, #K1622) following the manufacturer’s protocol. Quantitative real-time PCR was performed using a specific SYBR Green Gene Expression Assay (Applied Biosystems, #4309155) for the FYN gene and an endogenous control (ACTB) on a real-time PCR system. FYN: forward, 5′-CAG​TTG​ACT​CCA​TCC​AGG​CAG​A-3′, and reverse, 5′-CAC​GGA​TGG​AAA​GTG​AGT​AGG​C-3′.

PIK3R1: forward 5′-CGC​CTC​TTC​TTA​TCA​AGC​TCG​TG-3′, and reverse, 5′- GAA​GCT​GTC​GTA​ATT​CTG​CCA​GG-3′. ACTB: forward, 5′-CAT​TGC​TGA​CAG​GAT​GCA​GAA​GG-3′, and reverse, 5′-TGC​TGG​AAG​GTG​GAC​AGT​GAG​G-3′. Each reaction was run in triplicate. The relative expression levels of the FYN gene were calculated using the 2^(−ΔΔCt) method, where Ct represents the threshold cycle, and data were normalized to the endogenous control. The qPCR results presented in display the average RNA expression level of FYN and PIK3R1 across three mice.

#### 2.2.6 Western blot analysis

Cell lysates were prepared by washing cells with cold PBS, followed by lysis in RIPA buffer (Cell Signaling Technology, #9806) containing protease and phosphatase inhibitors. Protein concentrations were determined to use the BCA Protein Assay Kit (Thermo Fisher Scientific, #23225). Equal amounts of protein were separated by SDS-PAGE and transferred onto PVDF membranes (Millipore, #IPVH00010). The membranes were blocked with 5% non-fat milk in TBS-Tween for 1 h at room temperature and then incubated overnight at 4°C with primary antibodies against FYN (Cell Signaling Technology, #4023), PIK3R1 (Abcam, #ab32089) and GAPDH (Cell Signaling Technology, #5174) at the recommended dilutions. After washing, the membranes were incubated with the appropriate HRP-conjugated secondary antibodies for 1 h at room temperature. Protein bands were visualized using an enhanced chemiluminescence detection system (Thermo Fisher Scientific, #34076) and quantified with densitometry analysis software.

## 3 Results

### 3.1 Identification of potential Icaritin targets

**FIGURE 1:**
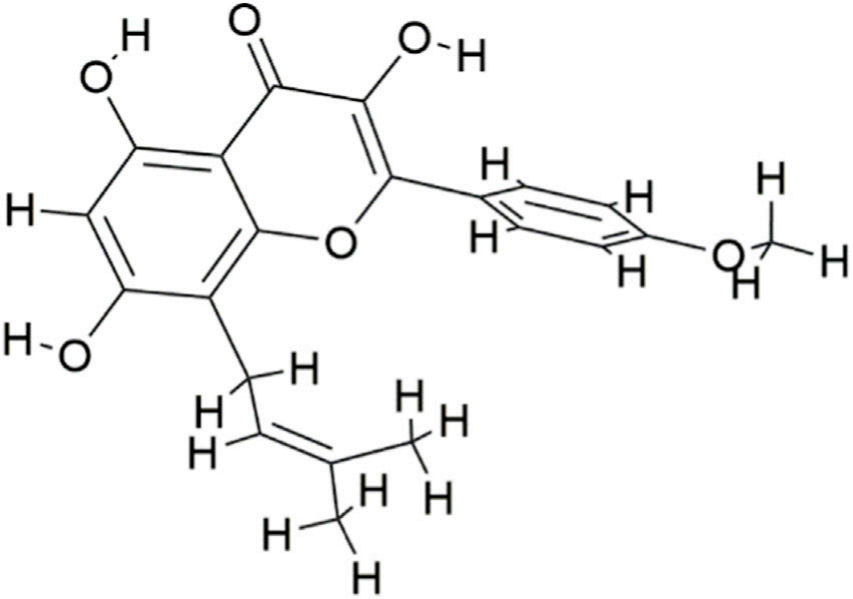
The chemical structure of Icaritin.

**FIGURE 2:**
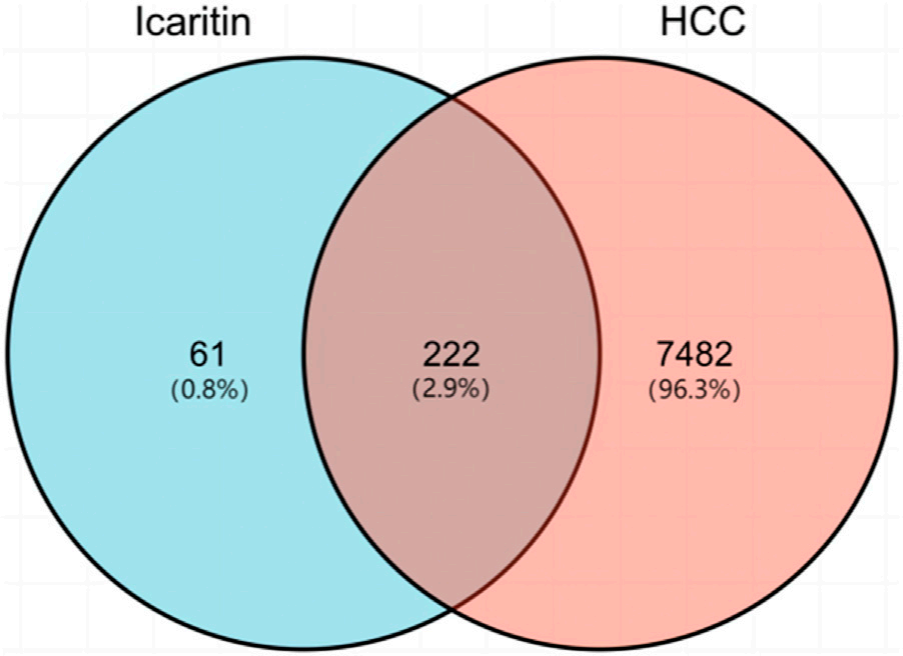
Venn diagram of Icaritin targets and HCC targets.

The molecular structure of Icaritin is presented in Figure 1. By employing a Venn diagram, 283 potential Icaritin targets and 7,704 hepatocellular carcinoma (HCC) targets were intersected, resulting in a total of 222 intersected targets (Figure 2). These targets represent the potential anti-HCC targets of Icaritin.

### 3.2 Construction of PPI network and identification of core Icaritin Anti-HCC targets

**FIGURE 3:**
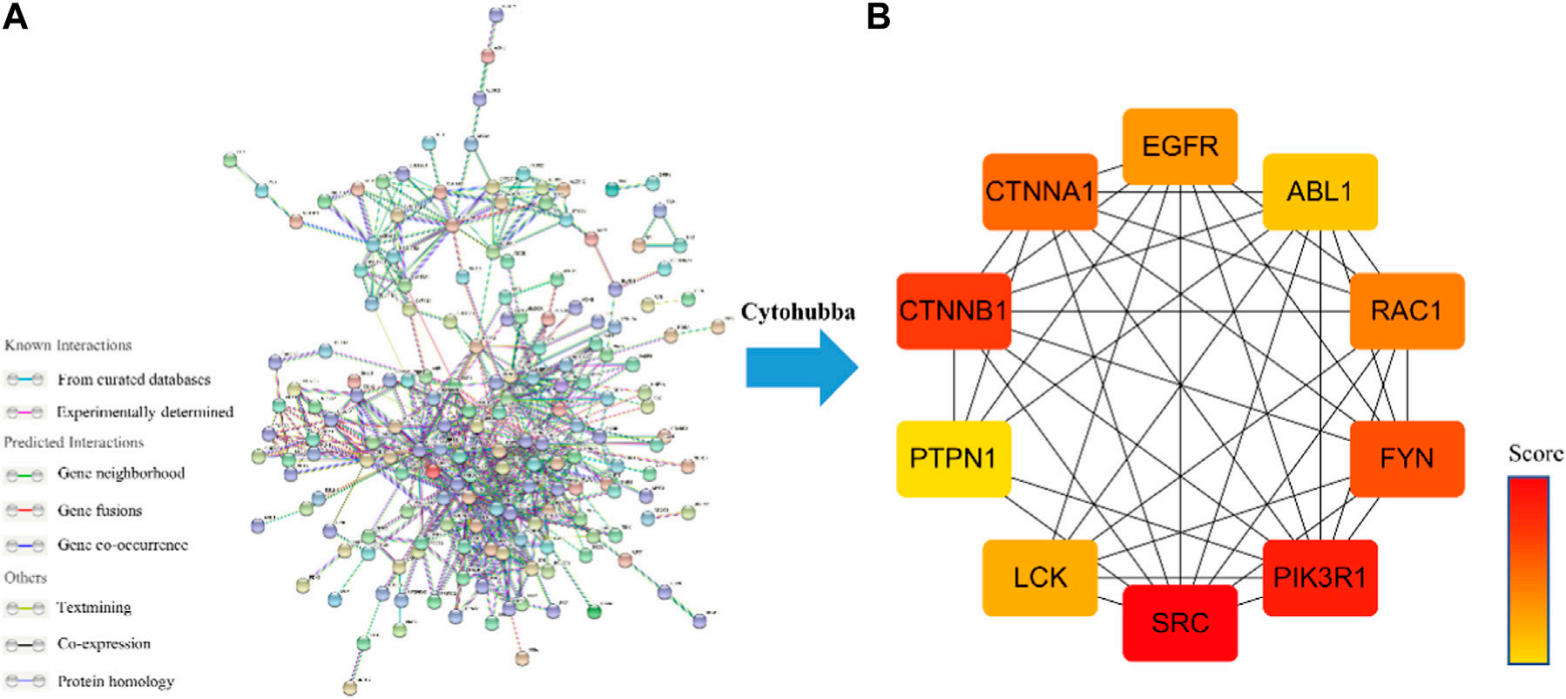
**(A)** Drug-component-disease-target network diagram. **(B)**The core targets for the anti-HCC of Icaritin.

The PPI network of Icaritin’s anti-HCC targets is illustrated in Figure 3A, containing 132 nodes and 554 edges. Utilizing the cytoHubba plugin in Cytoscape 3.7.2 with the Matthews Correlation Coefficient (MCC) method for screening, ten core targets were identified (Figure 3B), specifically SRC, PIK3R1, CTNNB1, FYN, CTNNA1, RAC1, EGFR, LCK, ABL1, and PTPN1. The MCC method is a network analysis approach that ranks nodes based on their connectivity and importance within the network. In this study, the core anti-HCC targets were selected based on their MCC scores, with higher scores indicating higher importance within the network. The core anti-HCC target scores for these genes, determined by the MCC method, are provided in Table 1.

**TABLE 1:** Core targets in network String Network ranked by MCC method.

Rank	Name	Score
1	SRC	5068
2	PIK3R1	4272
3	CTNNB1	3515
4	FYN	3342
5	CTNNA1	3150
6	RAC1	2882
7	EGFR	2302
8	LCK	1220
9	ABL1	1150
10	PTPN1	987

### 3.3 GO enrichment analysis of Icaritin’s anti-HCC targets

**FIGURE 4:**
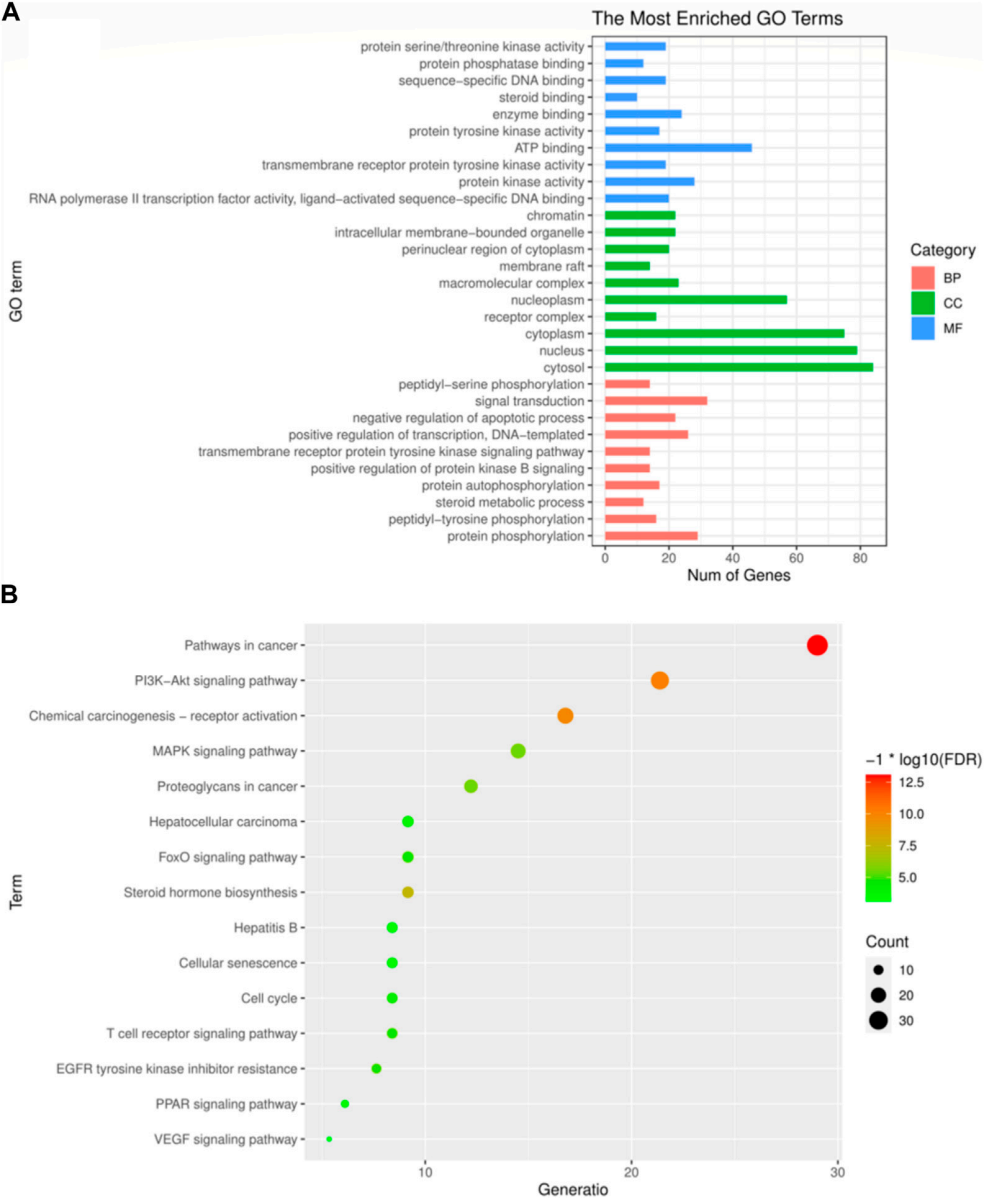
GO and KEGG enrichment analysis. **(A)** The top 30 most enriched GO categories and pathways were calculated and plotted. **(B)** Top15 KEGG pathways.

The principal biological processes of Icaritin’s anti-HCC targets involve protein phosphorylation, peptidyl-tyrosine phosphorylation, steroid metabolism, autophosphorylation of proteins, and positive regulation of the protein kinase B signaling pathway. The primary cellular components are the cytosol, nucleus, cytoplasm, receptor complex, and nucleoplasm. The molecular functions include RNA polymerase II transcription factor activity, ligand-activated sequence-specific DNA binding, activity of protein kinase, transmembrane recipient protein tyrosine kinase activity, and ATP binding. The GO enrichment analysis of Icaritin’s anti-HCC targets is visualized in Figure 4A.

### 3.4 KEGG pathway analysis of Icaritin’s Anti-HCC targets

The primary pathways through which Icaritin exerts its anti-HCC effects include cancer pathways, PI3K-Akt signaling pathway, MAPK signaling pathway, resistance mechanisms to inhibitors of the epidermal growth factor receptor tyrosine kinase, and the HCC pathway. These pathways suggest that Icaritin has therapeutic potential for HCC treatment. The KEGG pathway analysis of Icaritin’s anti-HCC targets is visualized in Figure 4B, with entry details provided in Figure 4.

### 3.5 Molecular docking

**FIGURE 5:**
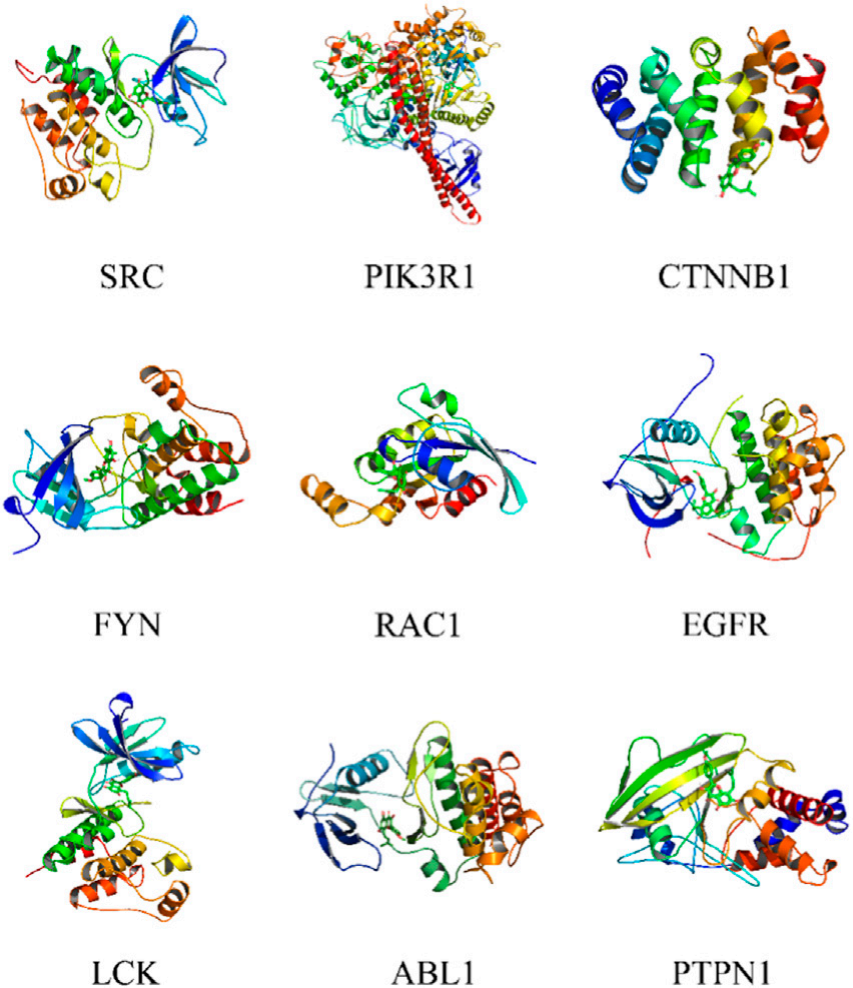
Molecular docking of Icaritin with core targets.

Icaritin was molecularly docked with nine core targets, excluding CTNNA1 due to its lack of a ligand-containing crystal complex structure. Binding energy data for these targets is displayed in Table 2. Lower binding energies indicate better docking results. Binding energies less than −5.0 kJ/mol suggest favorable binding, while those less than −7.0 kJ/mol indicate strong binding activity. Icaritin exhibited strong binding activity to FYN, EGFR, and LCK, as their binding energies were less than or equal to −8 kJ/mol. The molecular docking diagram for Icaritin and core targets is shown in Figure 5.

**TABLE 2:** Molecular docking results of Icaritin with core targets.

Target	PDB:ID	Affinity (KJ/mol)	Docking Site(x,y,z)
SRC	4u5j	−7.9	−9.141, 59.065, 40.674
PIK3R1	4ovv	−3.6	65.999, 40.928, 110.537
CTNNB1	7afw	−5.6	59.556, −40.342, 17.8
FYN	2dq7	−10.0	−16.501, 18.133, 13.115
RAC1	5qqd	−7.4	−3.817, 12.275, 30.726
EGFR	6s9c	−9.1	−21.764, −51.575, −0.863
LCK	2og8	−9.5	11.222, 5.775, 31.198
ABL1	4wa9	−6.9	8.21, 156.477, 36.573
PTPN1	7klx	−7.1	37.289, 31.776, 23.143

### 3.6 Molecular dynamics simulation

**FIGURE 6:**
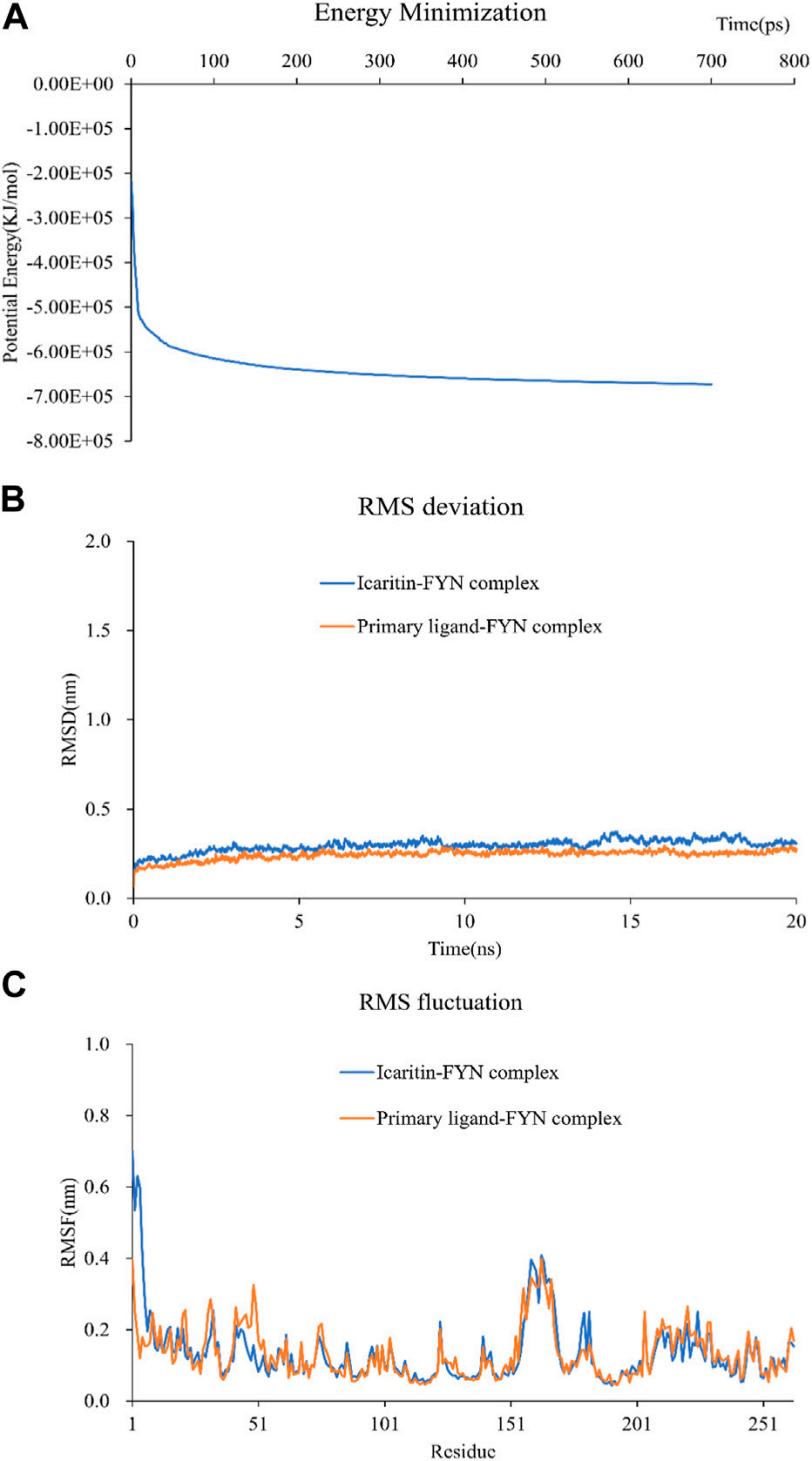
Molecular dynamics simulation of compound-target complex. **(A)** The energy minimization analysis of the molecular dynamics simulation of the icaritin-fyn docking complex. **(B)** The RMSD deviation analysis for the icaritin-FYN complex. **(C)** The RMSD fluctuation analysis for the icaritin-FYN complex.

Selection of Icaritin-FYN docking complexes for molecular dynamics simulations, as it had the lowest binding energy. These simulations supported the molecular docking findings and clarified the dynamic interactions between protein-ligand complexes. The system potential converges in the 700 ps range (Figure 6A), and the RMSD plot demonstrated the stability of the Icaritin-FYN complex throughout the simulations (Figure 6B). Meanwhile, the RMSF showed relatively limited fluctuations of protein residues (Figure 6C). Protein-ligand free energy calculations were conducted using MM/PBSA for the Icaritin-FYN complex and the FYN-primary ligand complex.

### 3.7 *In vivo* and *in vitro* experiments

#### 3.7.1 *In vivo* experiments

**FIGURE 7:**
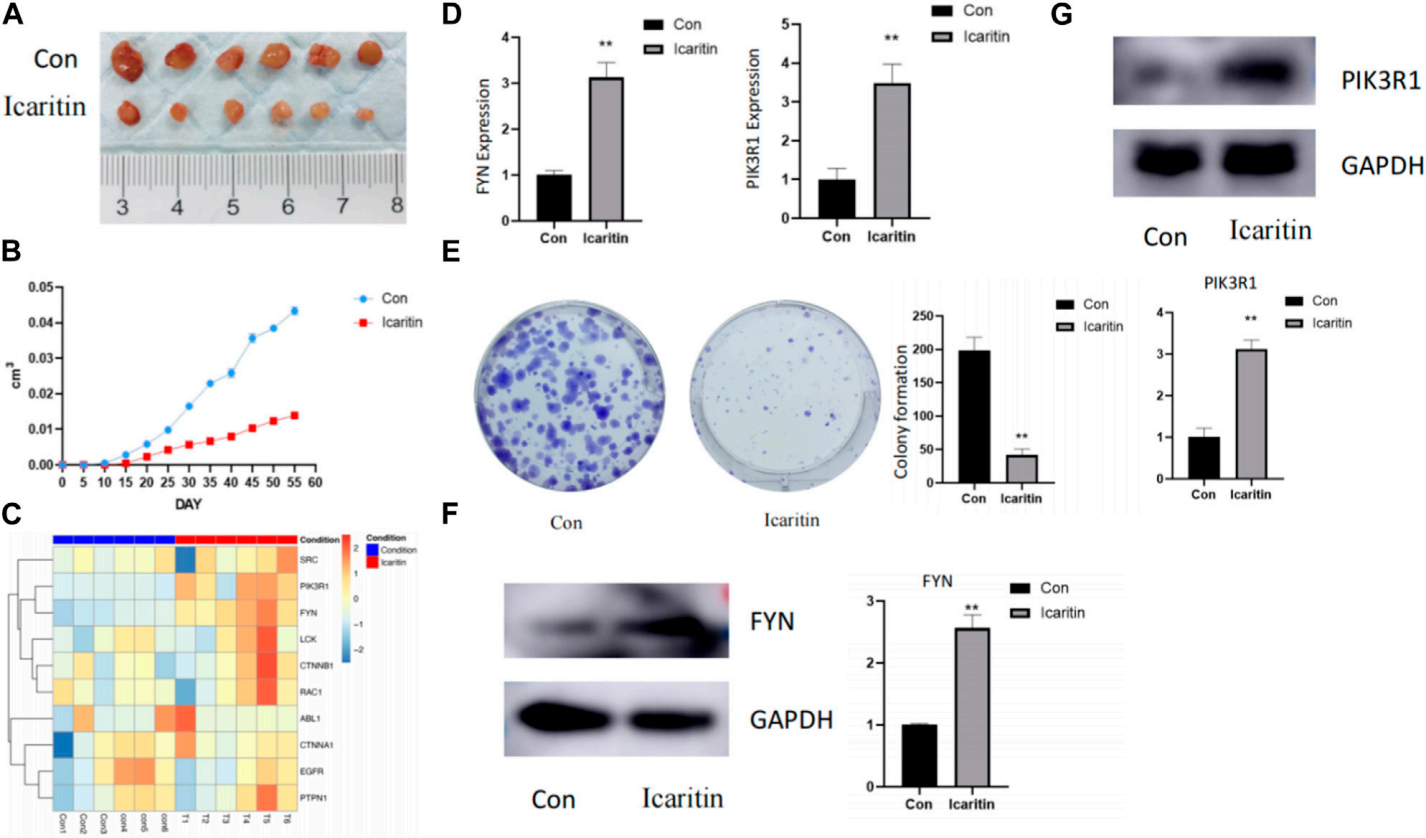
*In vivo* and *in vitro* experimental results. **(A)** Comparison of tumor size between Icaritin-treated and control groups in the animal study. Six-week-old male BALB/c nude mice were utilized (*n* = 6 per group). Mice were subcutaneously inoculated with HepG2 cells in the right flank region and randomly allocated into two groups: the Icaritin treatment group and the control group. The Icaritin treatment group received intraperitoneal injections of Icaritin at a concentration of 0.2 mg/kg Icaritin, while the control group was given injections of physiological saline. Injections were administered for 8 weeks until the study’s completion. Tumor progression and body weight were periodically assessed. At the end of the experiment, mice were euthanized, and tumor tissues were excised for subsequent analysis. A scale is included in the figure to show the tumor size difference. **(B)** Tumor growth curve in Icaritin-treated and control groups. **(C)** Heatmap of predicted Icaritin-targeted protein expression in the proteomics analysis, highlighting the significant difference in PIK3R1 and FYN protein expression between Icaritin-treated and control groups (*n* = 6 per group). **(D)** qPCR results showing a significant upregulation of PIK3R1 and FYN genes in the Icaritin-treated group compared to the control group (**, *p* < 0.01). **(E)** Colony formation images for Icaritin-treated and control HepG2 cells, demonstrating a significant reduction in colony numbers for the Icaritin-treated group (, *p* < 0.01). **(F)** and **(G)** Western blot results of PIK3R1 and FYN protein expression in Icaritin-treated and control HepG2 cells, showing a significant upregulation of PIK3R1 and FYN protein in the Icaritin-treated group (**, *p* < 0.01).

Figure 7A illustrates that tumors treated with Icaritin were clearly small for the control group. A targeted proteomics analysis was performed on tumors from both groups. Figure 7B presents a heatmap of the predicted Icaritin-targeted protein expression, which demonstrates a significant upregulation of FYN protein in the Icaritin-treated group with excellent repeatability. Furthermore, qPCR results depicted in Figure 7C reveal a significant upregulation of the FYN gene in the Icaritin-treated group.

#### 3.7.2 *In vitro* experiments

*In vitro* validation was carried out using HEPG2 cells. Figure 7D displays images of colony formation for Icaritin-treated and control groups, revealing that the number of colonies formed by Icaritin-treated cells was significantly lower compared to the control group. FYN protein expression differed considerably between the two groups, and FYN protein expression was significantly upregulated in the Icaritin group (Figure 7E).

## 4 Discussion

This study’s objective was to look into the potential anti-hepatocellular carcinoma (HCC) impacts of Icaritin and its underlying molecular mechanisms using a combination of *in vitro* and *in vivo* experiments, and network pharmacology and molecular docking approaches. Previous *in vitro* and *in vivo* studies have demonstrated that Icaritin exhibits strong inhibitory effects on the growth of HCC cells. However, the underlying mechanism of action requires further exploration (Liu et al., 2022; Wu, 2022). Network pharmacology, an innovative approach incorporating bioinformatics and network analysis, enables a systematic investigation of potential drug targets and molecular mechanisms at the systems level (Hao and Xiao, 2014; Nogales et al., 2022). The possible targets and molecular mechanisms of Icaritin against HCC were carefully examined for this study using a network pharmacology approach. We discovered important targets and pathways implicated in Icaritin’s putative anti-HCC action by network pharmacology research. Our findings showed that Icaritin may exert its anticancer effects through processes related to phosphorylation, steroid metabolism, the PI3K-Akt signaling pathway, the MAPK signaling pathway, and resistance to tyrosine kinase inhibitors of the receptor for epidermal growth factor (Yang et al., 2019). These pathways play critical roles in cell survival, proliferation, differentiation, and tumorigenesis. We further identified ten core targets (SRC, PIK3R1, CTNNB1, FYN, CTNNA1, RAC1, EGFR, LCK, ABL1, and PTPN1) that may mediate Icaritin’s anti-HCC action. These targets are participated in various cellular processes, including cell signaling, adhesion, migration, invasion, and proliferation. Including HCC, dysregulation of these targets has been linked to the emergence of a variety of cancer forms. Among these core targets, FYN showed the highest molecular docking binding energy with Icaritin, suggesting that FYN might play a crucial role in Icaritin’s anti-HCC activity. The molecular docking and subsequent molecular dynamics simulations further verified the interactions between Icaritin and the core targets. The higher score of PIK3R1 was found by cytohubba calculation, indicating that PIK3R1 is at the core of the PPI network and cannot really indicate that there is an interaction between the proteins. In contrast, the results of molecular docking, which is a docking interaction between epimedin and core proteins, showed that epimedin has higher docking binding energy to other targets compared to PIK3R1. The two are not equivalent. Specifically, FYN demonstrated strong binding activity with Icaritin, and the obtained complexes have higher binding free energy than the original ligand-FYN complexes. These findings indicate that FYN is likely an essential target for Icaritin’s action against HCC.

A proto-oncogene from the Src family called FYN has been found in several investigations to increase cancer cell proliferation and prevent apoptosis. FYN is essential for mitogenic signaling, cell cycle entry, expansion, and proliferative processes (Gururajan et al., 2015). Both the mRNA and protein levels of FYN are upregulated in thyroid cancer, which promotes cell growth and inhibits apoptosis (Zheng et al., 2017). Since microRNA-125a-3p specifically targets FYN, it causes FYN downstream proteins to be produced and reduced FYN expression and function, hence inhibiting cell division. This indicates that FYN stimulates tumor cell growth (Ninio-Many et al., 2013). Increased FYN expression and activity in chronic granulocytic leukemia enhances the transition into the acute period and stimulates cell growth (Singh et al., 2012). In glioblastoma, FYN phosphorylates PIKE-A, increasing its binding to AMPK, lowering AMPK’s tumor suppressive function, and stimulating tumor cell growth (Zhang et al., 2016). FYN inhibition reduces the growth of pancreatic cancer cells (Je et al., 2014). Several clinical trials are presently ongoing to explore FYN/SRC inhibitors, which not only increase chemotherapy effectiveness (Ma et al., 2019; Wang et al., 2022), but also improve immunotherapy and radiation therapeutic response (Yun et al., 2021; Kadota et al., 2022).

Results from the *in vivo* investigation showed that Icaritin significantly decreased tumor size in the treatment group compared to the control group (Figure 7A). This reduction in tumor size can be ascribed to the possible anti-proliferative, anti-angiogenic, and pro-apoptotic effects of Icaritin. In the context of the targeted proteomics analysis, a significant upregulation of FYN protein was observed in the Icaritin-treated group (Figure 7B). This discovery implies that FYN could be essential in the anticancer activity of Icaritin against HCC. FYN is a member of the Src family kinases, which have been implicated in various cancer types, including breast, lung, colon, and prostate cancer, among others. Recent research on the FYN gene has revealed its involvement in cancer progression, metastasis, and drug resistance. Moreover, FYN has been identified as a potential therapeutic target for cancer treatment due to its roles in tumor cell proliferation, survival, and invasion. As such, the development of new drugs targeting FYN could hold significant promise for cancer therapy.

The *in vitro* experiments conducted on HEPG2 cells provided further evidence for the anticancer activity of Icaritin. The Icaritin-treated cells exhibited significantly reduced colony formation compared to the control group (Figure 7D), indicating a potential anti-proliferative effect of Icaritin on HCC cells. Furthermore, a marked difference in FYN protein expression was detected between the Icaritin-treated and control groups, with the Icaritin group showing a significant upregulation of FYN protein (Figure 7E). This finding reinforces the importance of FYN in the anticancer mechanism of Icaritin against HCC.

Finally, the results of this investigation showed that Icaritin exhibits significant anticancer effects against HCC both *in vivo* and *in vitro*, possibly through the modulation of FYN protein expression. These findings highlight the potential therapeutic uses of Icaritin in the treatment of HCC and help to better understand the molecular processes behind the anti-HCC action of the substance. Future studies should focus on investigating the precise signaling pathways involved in the Icaritin-mediated regulation of FYN, as well as assessing the efficacy and safety of Icaritin in preclinical and clinical settings (Kim et al., 2010).

## Data Availability

The original contributions presented in the study are included in the article/supplementary material, further inquiries can be directed to the corresponding authors.
